# Glycosaminoglycans and Glycosaminoglycan Mimetics in Cancer and Inflammation

**DOI:** 10.3390/ijms20081963

**Published:** 2019-04-22

**Authors:** Shravan Morla

**Affiliations:** 1 Department of Medicinal Chemistry, Drug Discovery and Development, Virginia Commonwealth University, Richmond, VA 23219, USA; morlas@vcu.edu; 2 Institute for Structural Biology, Drug Discovery and Development, Virginia Commonwealth University, Richmond, VA 23219, USA

**Keywords:** glycosaminoglycans, mimetics, heparin, heparan sulfate, chondroitin sulfate, dermatan sulfate, hyaluronan, keratan sulfate, anti-cancer, anti-inflammation

## Abstract

Glycosaminoglycans (GAGs) are a class of biomolecules expressed virtually on all mammalian cells and usually covalently attached to proteins, forming proteoglycans. They are present not only on the cell surface, but also in the intracellular milieu and extracellular matrix. GAGs interact with multiple ligands, both soluble and insoluble, and modulate an important role in various physiological and pathological processes including cancer, bacterial and viral infections, inflammation, Alzheimer’s disease, and many more. Considering their involvement in multiple diseases, their use in the development of drugs has been of significant interest in both academia and industry. Many GAG-based drugs are being developed with encouraging results in animal models and clinical trials, showcasing their potential for development as therapeutics. In this review, the role GAGs play in both the development and inhibition of cancer and inflammation is presented. Further, advancements in the development of GAGs and their mimetics as anti-cancer and anti-inflammatory agents are discussed.

## 1. Introduction

**Figure 1:**
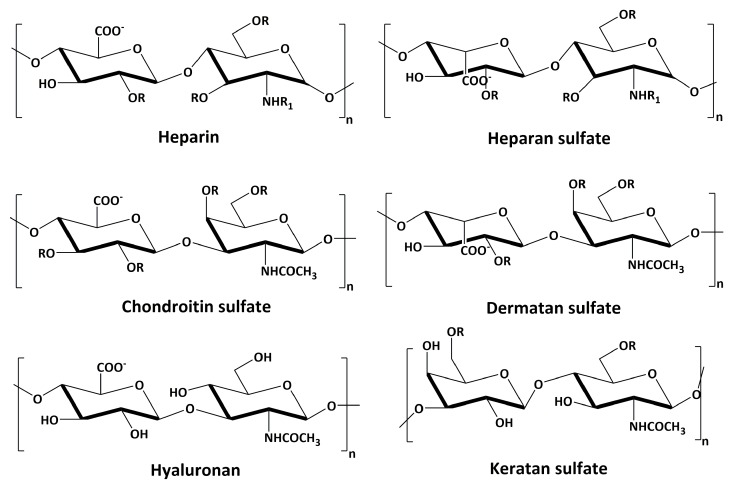
Major disaccharide repeating units of different types of GAGs (R = H or SO_3_^−^ and R_1_ = H, or SO_3_^−^, or COCH_3_^−^).

Glycosaminoglycans (GAGs) are linear polysaccharides whose disaccharide building blocks consist of an amino sugar (d-glucosamine that is *N*-acetylated, or *N*-sulfated, or *N*-acetyl-d-galactosamine) and either uronic acid (d-glucuronic acid or L-iduronic acid) or galactose. GAGs are sometimes referred to as mucopolysaccharides as they were originally characterized in mucus membranes and mucosal exudates. GAGs are differentiated from one another based on the type of monomeric unit, linkages between each monomeric unit, the position of sulfate groups, and the degree of sulfation. Based on these features, GAGs can be broadly classified into four different classes (Figure 1): chondroitin sulfate/dermatan sulfate (CS/DS), heparin/heparan sulfate (HS), hyaluronan (HA), and keratan sulfate (KS) [1].

GAGs are highly charged owing to negatively-charged carboxylic acid units present on the uronic acid residues and sulfate groups present on most of the units. HA is the only GAG that is not sulfated and, hence, is the least negatively-charged GAG, while heparin is the most negatively charged. Although each of these GAGs has a predominant disaccharide component as mentioned above, as a result of the non-template-driven biosynthetic pathway by which they are produced, these disaccharides can be modified at multiple positions by sulfation, acetylation, and/or epimerization, creating a tremendous amount of heterogeneity in any particular class of GAGs [2]. 

GAGs are involved in a myriad of biological functions. Most of the biological interactions mediated by proteoglycans are believed to be primarily because of the GAG chains present on their surface. Since GAGs make up the cell surface of almost all the cells in the body, they play a major role in maintaining the structural integrity of cells and tissues. They bind to different protein targets, primarily via electrostatic interactions between negatively-charged uronic acids and sulfate groups and positively-charged amino acids in the protein. In addition to electrostatic interactions, non-ionic interactions between GAGs and their binding partners are found to dictate the specificity and selectivity of binding [3]. They are known to interact with cytokines, chemokines, growth factors, and enzymes, leading to profound physiological effects on processes such as coagulation, growth, infection, inflammation, tumor progression, metastasis, etc. (Table 1). 

Since GAGs are involved in a plethora of biological activities, their use in the development of drugs has been of long interest in the pharmaceutical industry. Heparin, the first GAG-based drug, is used as an anticoagulant for the treatment of thrombosis, thrombophlebitis, and embolism [14]. The therapeutic potential of GAGs and their mimetics for the treatment of many other diseased states, including cancer, inflammation, infection, wound healing, lung diseases, Alzheimer’s disease, etc., are being actively studied [15]. In this review, however, only the role and therapeutic application of GAGs and GAG mimetics in cancer and inflammation will be discussed. 

## 2. Role of GAGs in Cancer

Interaction of GAGs with growth factors, growth factor receptors, and cytokines are implicated in cancer growth, progression, and metastasis [16]. GAGs are found to be involved in multiple signaling cascades required for angiogenesis, cancer invasion, and metastasis. Interestingly, some GAGs have also been shown to play a role in the inhibition of tumor progression. Understanding the type of interaction and the role these GAGs play in multiple cancer types has led to the development of various therapeutic approaches and novel drugs to treat cancer [17]. 

### 2.1. Heparin/HS in Cancer

Heparan sulfate proteoglycans (HSPGs) through their core proteins and GAG side chains modulate multiple functions of tumor cells and are involved in tumor growth, invasion, and metastasis [18,19]. HS has been shown to promote cell-cell and cell-ECM adhesion, inhibiting invasion and metastasis, and a decrease in the levels of HS, as seen in some cancers, resulted in the malignant cells being more invasive [7,20]. While the levels of HS are decreased in some cancers, others display modifications in their sulfation pattern, which are shown to be responsible for cancer progression. For example, highly-sulfated HS is shown to trigger cell proliferation through fibroblast growth factor 2 (FGF2) signaling, in melanoma cells [8]. Additionally, in the case of colon cancer, these modifications were also found to be highly dependent on the anatomical location (right-side vs. left-side) and the metastatic/non-metastatic nature of the tumor. Surprisingly, metastatic right-sided colorectal cancers were found to exhibit fewer changes to the structure of HS when compared to the non-metastatic tumors [21,22]. HS also plays a crucial role in epithelial to mesenchymal transition (EMT) due to its ability to bind to growth factors present in the tumor microenvironment [23].

HSPGs, such as syndecan-1 and syndecan-4, have been associated with breast cancer progression by the formation of complexes with FGF2 and fibroblast growth factor receptor 1 (FGFR-1) [24]. It was also shown that syndecan-4 expression promotes tumor adhesion and migration in melanoma cells [25]. Another class of HSPGs, glypican-3, is found to play a variable role in tumors. For example, it is shown to promote tumor growth in hepatocellular carcinoma and melanoma by stimulating Wnt signaling [26], whereas it inhibited tumor growth by inducing apoptosis in breast and ovarian cancers [18]. However, glycosylphosphatidylinositol-anchored glypicans, when overexpressed, are associated with tumor growth in hepatocellular carcinoma and melanoma.

Over-expression of heparanase (HPSE), an enzyme that cleaves HS chains of HSPGs at β-1,4 positions, has been shown to be involved in mechanisms promoting tumor growth, angiogenesis, and metastasis [27,28,29]. HPSE also enhances the phosphorylation of Src and epidermal growth factor receptor (EGFR), activating STAT3, which is associated with head and neck cancer progression [30]. It further stimulates an increase in the expression of both hepatocyte growth factor and syndecan-1, resulting in an aggressive phenotype [31]. On the contrary, heparanse-2 inhibits the activity of HPSE, thereby acting as a tumor suppressor [32].

Downregulation of human sulfatase-1 (SULF1), a heparin-degrading endosulfatase, observed in breast cancer cells, is shown to increase cell migration and invasion [33]. Human sulfatase-2 (SULF2), an HS 6-*O*-endosulfatase, was found to inhibit in vivo tumor growth in human breast cancer xenograft models [34]. Conversely, in the case of lung cancer, it is shown to promote carcinogenesis [35].

### 2.2. CS/DS in Cancer

CS/DS is involved in the regulation of critical cellular processes, such as proliferation, apoptosis, migration, adhesion, and invasion [36]. The CS/DS side chains of chondroitin sulfate proteoglycans (CSPGs) participate in various interactions within the ECM, which is of particular importance in malignancy. For example, increased levels of versican, a CSPG, has been correlated with disease progression in early-stage breast and prostate cancers [37,38]. Changes to the CS chains of versican with enhanced expression of 6-*O*-sulfated and non-sulfated disaccharide units was observed in a malignant phenotype of pancreatic cancer [39]. In the case of melanoma, increased levels of melanoma-associated chondroitin sulfate proteoglycan (MCSP) are observed, which enhances integrin function, thereby activating Erk1/2 and stimulating cell growth and motility [40].

**Figure 2:**
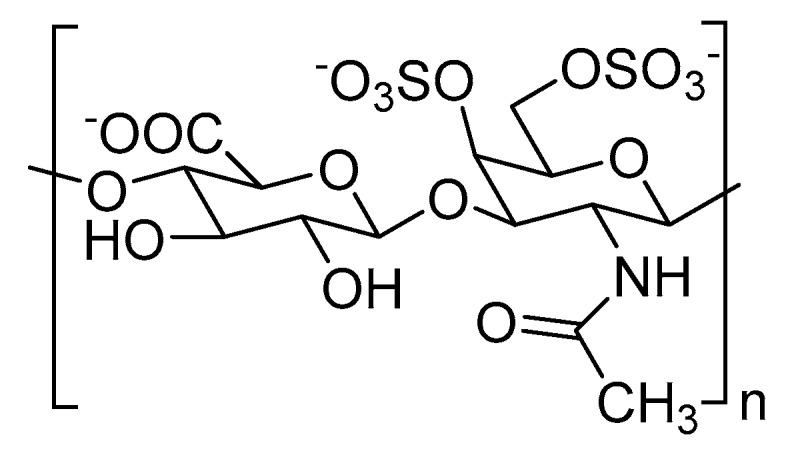
Chemical structure of chondroitin-4,6-disulfate or CS-E.

Structural alterations to CS chains, especially their sulfation pattern, is associated with cancer progression [41]. For example, chondroitin-4,6-disulfate or CS-E (Figure 2), a type of CS, is overexpressed in multiple cancer types. High expression of CS-E is correlated with increased binding to vascular endothelial growth factor (VEGF) in ovarian adenocarcinomas [42]. A higher proportion of CS-E was found in the highly-metastatic LM660H11 lung carcinoma cell line, when compared to the P29 cell line with low metastatic potential, suggesting the role of CS-E in metastasis [43]. It was also shown that overexpression of CS-E causes colonization of osteosarcoma cells in the liver [44].

Changes in the expression levels of CS biosynthetic enzymes are also found in certain cancers [21,22]. Chondroitin sulfate *N*-acetylgalactosaminyltransferase 2 (CSGALNACT2), which plays a critical role in chain elongation during CS synthesis, is found to be downregulated in non-metastatic colorectal cancers, both right and left [21]. Similar to HSPGs, these changes are also dependent on whether or not a tumor is metastatic [21].

CS/DS are also shown to interact with the proteins involved in tumor growth and enhance their activity [45]. For example, CS-A, in human fibrosarcoma cells, enhances the mitogenic activity of platelet-derived growth factor-BB (PDGF-BB), which is involved in the growth of malignant cells and angiogenesis. The presence of CS-A increases the efficiency of signaling between PDGF-BB and tyrosine kinases. It was also observed that exogenous addition of CS-A enhances fibrosarcoma cell adhesion, chemotaxis, and migration [46].

### 2.3. HA in Cancer

Increased levels of HA are found in multiple types of human cancers, including breast [47], lung [48], and ovarian cancer [49]. Growth factors and chemokines present in the tumor microenvironment are known to induce HA production [50]. On the other hand, low levels of HA are associated with metastatic potential in squamous cell carcinoma [51] and melanoma [52].

CD-44, in the presence of high levels of HA, is found to interact with signaling receptors such as EGFR to stimulate downstream pathways involving PI3k/Akt and/or mitogen-activated protein (MAP) kinases promoting chemoresistance and breast cancer progression [53,54,55]. Moreover, degradation of HA to smaller oligosaccharides by hyaluronidases is reported to induce cleaving of CD-44 in the tumor microenvironment, leading to tumor progression, as seen in breast, ovarian, glioma, and colon cancers [56,57].

### 2.4. GAGs as Anti-Cancer Agents

Several researchers have worked on utilizing the involvement of GAGs in cancer to develop therapeutics. For example, heparin, in addition to its anticoagulant properties, also possesses anti-cancer properties [58,59]. However, its anticoagulant property limits its development as an anticancer agent. Taking this into consideration, non-anticoagulant heparin analogs inhibiting heparanase in vitro and in vivo have been developed [60,61,62].

A non-anticoagulant HS, isolated from the bivalve mollusk *Nodipecten nodosus*, has been shown to inhibit P-selectin-mediated events such as metastasis of Lewis lung carcinoma cells [63]. Another modified non-anticoagulant heparin, SST0001 or Roneparstat, was developed by Sigma-Tau Pharmaceuticals as a potent heparanase inhibitor [61]. SST0001 is 100% *N*-acetylated and 25% glycol split high-molecular weight heparin, and hence, the microheterogeneity of the original heparin is retained in it. In preclinical murine models, it showed a significant reduction in tumor volume in multiple myeloma mice xenograft models and was also recently tested in Phase I clinical trials for advanced multiple myeloma [61].

M402 or necuparanib is another molecule developed as a non-anticoagulant heparin [60]. It is obtained from depolymerization of low molecular weight heparin followed by oxidation and borohydride reduction, resulting in a glycol split. It was found to inhibit multiple targets involved in tumor progression and metastasis, including heparanase, VEGF, FGF2, P-selectin, and stromal cell-derived factor-1α (SDF-1α). It showed a reduction in tumor metastasis and statistically-significant survival benefits in preclinical studies [64]. However, the Phase II clinical study in combination with nab-paclitaxel and gemcitabine for pancreatic cancer was terminated due to an insufficient level of efficacy in the study population.

Desai and co-workers found that a particular non-anticoagulant hexasaccharide sequence of HS, called HS06, selectively inhibited cancer-stem-cell (CSC) self-renewal and induced apoptosis in several colorectal, breast, and pancreatic cell lines [65]. The inhibition of self-renewal was found to be because of activation of p38α/β mitogen-activated protein kinase (MAPK), which led to inhibition of TCF4 signaling, a critical regulator of CSC self-renewal. Additionally, it was also found that either shorter or longer saccharide sequences of HS were not able to inhibit CSCs as potently as HS06, portraying the specificity of GAG–protein interactions.

Sasisekharan and coworkers utilized the differential substrate specificity of heparinase I (Hep I) and heparinase III (Hep III) to generate different oligosaccharide GAG fragments from the tumor cell surface [66]. Interestingly, the fragments generated from Hep I promoted tumor growth, whereas Hep III generated fragments were found to inhibit tumor growth and metastasis. Further analysis showed that these GAG fragments inhibited tumor growth in vivo in a dose-dependent fashion with >70% reduction in primary tumor growth at a dose of 10 µg/kg/day in mice models.

Apart from heparin/HS GAGs, CS/DS-based targeting in cancer has also been widely studied. Fucosylated CS (FucCS), isolated from sea cucumber, was found to inhibit tumor metastasis in vivo by blocking P- and L-selectin-mediated events [67]. Exogenous CS-A has been shown to be effective in decreasing monocyte migration, thereby preventing angiogenesis in vitro [68]. Novel molecules called neoglycans, produced by modifying CS chains with carbodiimide, reduced tumor growth in nude mice with breast cancer without apparent toxicity to the normal tissue [69]. These studies prove the potential use of GAGs or modified GAGs as potent anticancer agents.

## 3. Role of GAGs in Inflammation

Inflammation is a defense mechanism of the body to harmful stimuli. This protective action primarily involves the recruitment of immune cells from the bloodstream into the site of injury/infection. Some of the major events during the inflammation process are intimately regulated by GAGs, especially those coating the surface of endothelial cells and leukocytes [70,71,72,73,74,75]. At the site of injury/infection, GAGs are involved in leukocyte rolling along the endothelial surface; regulation of chemokine migration and activation; and trans-endothelial migration of leukocytes.

Upon inflammatory trigger, macrophages at the site of infection/injury release cytokines, which activate endothelial cells, resulting in the display of P-selectins on their surface. P-selectin glycoprotein ligand-1 (PSGL-1) on leukocytes interact with activated endothelial cells by binding to P-selectins. The PSGL-1-P-selectin interaction enables leukocytes’ adhesion onto the endothelial layer [70]. Upon leukocyte adhesion on the endothelial cells, HS on the surface of endothelial cells binds to L-selectins on leukocyte, leading to leukocyte rolling [71]. Regulation of leukocyte rolling is the first role of GAGs in inflammation.

Macrophages at the site of infection/injury also release significant amounts of chemokines, in addition to cytokines. The released chemokines, particularly IL-8 (CXCL-8), bind to GAGs at the endothelial surface, leading to chemokine transcytosis [72]. After transcytosis across the endothelial layer, leukocytes are recruited by the interaction of integrin on leukocytes with intercellular adhesion molecule-1 (ICAM-1) on the endothelial surface. This interaction leads to increased adhesion of leukocytes onto the endothelium, thereby slowing down the rolling of leukocytes. This triggers morphological changes required for the migration of leukocytes through the endothelial barrier. Syndecan, a proteoglycan containing HS chains, plays a major role in this migration process [73].

Heparin is synthesized and stored in certain types of mast cells and is co-released with histamine upon inflammatory trigger [74]. Released heparin induces the formation of bradykinin and helps in vascular permeability [75].

### GAGs as Anti-Inflammatory Agents

Due to the tremendous role played by GAGs throughout the inflammation process, exogenous sulfated glycans of various structures can be used to downregulate inflammation processes. Many such molecules have been tested in in vitro and in vivo models, and some have even reached clinical trials.

Besides its use as an anticoagulant, heparin demonstrated excellent anti-inflammatory properties in animal models and clinical trials [76]. Heparin has been shown to be useful for the treatment of bronchial asthma [77], ulcerative colitis [78], and burns [79]. Although these studies showed promising results, heparin is not approved for use as an anti-inflammatory agent because of the associated bleeding risk. Several non-anticoagulant heparin mimetics were developed thereafter to maintain its anti-inflammatory effect [80]. One such example is 2-*O*,3-*O*-desulfated heparin (ODSH), which was shown to reduce airway inflammation by potently inhibiting human neutrophil elastase without any anticoagulant effects [81]. Another non-anticoagulant heparin isolated from shrimp *Litopenaeus vannamei* has significantly reduced the influx of inflammatory cells to the site of injury in acute inflammation models [82].

Exogenous DS of a specific length is found to inhibit P-selectins in inflammatory mouse models [83]. On the other hand, CS is found to inhibit inflammation in rat astrocytes by preventing NF-κB activation [84].

KS has been shown to ameliorate the pathological conditions associated with inflammation [85]. For example, exogenously-added KS reduced damage in cartilage explants that were exposed to interleukin-1α ex vivo. Since cartilage fragments can cause an antigenic response, resulting in an increase in inflammation and arthritic response, reduced cartilage degradation can be correlated to a reduction in the severity of arthritis [86]. In addition, when tested in vivo using a murine arthritis model, KS was found to ameliorate arthritis [86]. Plasma levels of KS have been identified as a potential biomarker for joint damage in juvenile idiopathic arthritis [87]. In the cornea, KS proteoglycans are found to bind to chemokine CXCL1 and facilitate its migration into the stroma during inflammation [88]. The addition of low molecular weight KS resulted in the disruption of this KS-CXCL1 complex, leading to efflux of chemokines and resolution of inflammation [89]. In a study by Taniguchi and coworkers, a KS disaccharide, [SO_3_^−^-6]Galβ1-4[SO_3_^−^-6]GlcNAc, prevented neutrophil-mediated inflammation and progression of emphysema in murine models, indicating its potential use for the treatment of inflammation in chronic obstructive pulmonary disease [90,91].

These works clearly indicate the potential of using GAGs and related compounds as anti-inflammatory agents.

## 4. GAG Mimetics

Although GAGs have tremendous applications as therapeutics, there are many challenges associated with their structure, halting their success in clinical trials. As previously mentioned, GAGs are complex heterogeneous molecules with exceptional structural diversity, which not only differ in their length, but are also modified at multiple positions through sulfation, acetylation, and epimerization. This inherent heterogeneity involved in the biosynthesis of GAGs leads to a particular GAG binding to many different proteins, thus compromising selectivity and leading to side-effects when given as a therapeutic [16,92].

Furthermore, GAGs are usually obtained from animal sources. For example, heparin, one of the oldest drugs in the clinic, is obtained from porcine intestine, bovine intestine, and bovine lung. Hence, the quality of heparin obtained depends on the environmental conditions and the diet each animal is exposed to and results in significant batch-to-batch variation [93]. The heterogeneity of GAGs makes the complete characterization of every batch of heparin produced nearly impossible, thereby making quality control a daunting task [94]. In 2008, contamination of heparin with over-sulfated CS resulted in over 200 deaths and thousands of adverse effects in the United States alone [95].

To address the issues involved in the development of GAGs as therapeutics, multiple strategies have been developed to mimic GAGs through small molecules called GAG mimetics [92]. GAG mimetics have numerous advantages over GAGs as therapeutics. They are usually completely synthetic and homogenous molecules and hence are expected to have increased selectivity and fewer adverse effects [96]. They are easier to produce at large scales, design computationally, characterize, and quality control. They also have better pharmacokinetic features than GAGs, making them more ‘drug-like’.

GAG mimetics can be classified into two classes: saccharide-based and non-saccharide-based. Saccharide-based GAG mimetics, although built on a sugar backbone, are synthetic and not produced from animal sources. They are less heterogeneous when compared to GAGs. On the other hand, non-saccharide-based mimetics utilize non-sugar-based scaffolds carrying negative charges through sulfates, sulfonates, carboxylates, and/or phosphates. They are completely homogenous molecules and provide numerous advantages over saccharide-based mimetics. Both saccharide and non-saccharide GAG mimetics have been developed for the treatment of cancer and inflammation, and a few are currently in clinical trials, while some are marketed in the clinic. Here, I discuss the GAG mimetics that have shown remarkable potential and made huge advancements in the fields of cancer and inflammation.

### 4.1. GAG Mimetics as Anti-Cancer Agents

#### 4.1.1. Saccharide-Based GAG Mimetics

**Figure 3:**
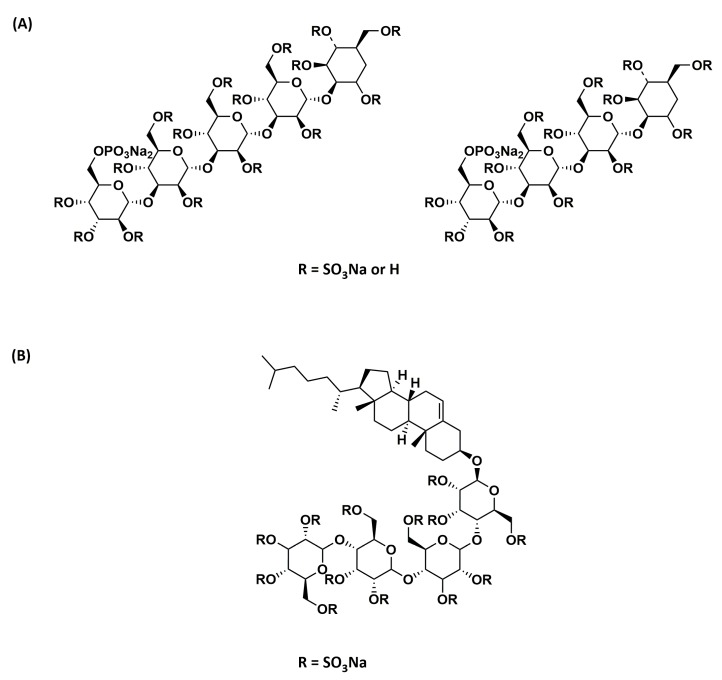
Chemical structure of (**A**) PI-88 and (**B**) PG545.

Phosphomannopentaose sulfate (PI-88; Figure 3A) is an HS mimetic obtained via sulfation of the phospho-mannan complex produced from yeast cultures [97]. It is a heterogeneous mixture of di- to hexa-saccharides, but mostly tetra- (60%) and penta-saccharides (30%). PI-88 potently inhibits the activity of heparanase, an enzyme that plays a vital role in metastasis and angiogenesis. It was also found to bind to pro-angiogenic growth factors VEGF, FGF1, and FGF2 by competing with HS. Although PI-88 also possesses anticoagulant activity, in addition to anticancer activity, it appeared to be well tolerated in preclinical models and was hence investigated further in clinical trials. It reached Phase III clinical trials for hepatocellular carcinoma, which was concluded after interim analysis due to the failure to reach the primary disease-free survival endpoint [98].

Several second-generation analogs of PI-88 that are anomerically pure, completely sulfated, and homogeneous were later developed by Progen Pharmaceuticals Ltd. Among these analogs, PG545 or pixatimod (Figure 3B) was selected as the lead compound [99]. In addition to being a fully-sulfated, anomerically-pure, homogenous compound, PG545 has a lipid moiety attached at the reducing end, which resulted in improved pharmacokinetic properties and reduced anticoagulant activity [100]. It also resulted in increased affinity towards heparanase as a result of the lipid moiety’s binding in the hydrophobic pocket [101]. It showed potent antitumor and anti-metastatic activity in several preclinical models and is currently in Phase I clinical trials for advanced solid tumors and metastatic pancreatic cancer.

Dollé and coworkers synthesized an octasaccharide heparin mimetic that interfered with the process of angiogenesis and metastasis [102]. It potently inhibited heparanase, FGF2, VEGF, SDF-1α, PDGF-β, and cell proliferation in vitro and is being tested in vivo in animal models.

#### 4.1.2. Non-Saccharide GAG Mimetics

**Figure 4:**
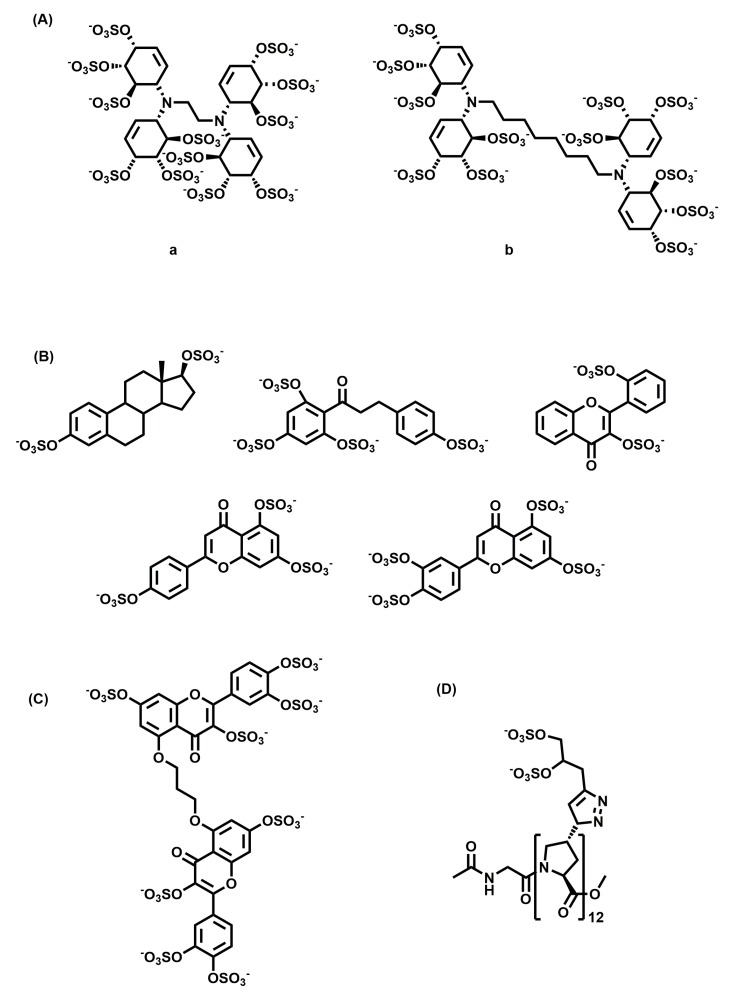
Chemical structures of non-saccharide glycosaminoglycan mimetics developed as anti-cancer agents: (**A**) sulfated cyclitols inhibiting growth factors, (**B**) angiogenesis inhibitors, (**C**) G2.2, a CSC inhibitor, and (**D**) {Z}_12_, a metastasis inhibitor.

A library of small HS mimetics was developed by Parish and co-workers from sulfation of cyclitols [103]. They screened the library of 15 molecules against a panel of GAG binding proteins and observed a clear relationship between the structure and the protein they recognized. They found that Compound **a** (Figure 4A) with an ethyl linker was a potent inhibitor of FGF-1, and Compound **b** (Figure 4A) with an octyl linker was a potent inhibitor of FGF-2 and VEGF, but not FGF-1. These compounds, because of their ability to inhibit different growth factors, are expected to possess antitumor activity.

Desai and co-workers reported the development of HS mimetics that inhibit angiogenesis [104]. By screening a library of 18 sulfated-small molecules belonging to different chemical classes (flavone, flavan, chalcone, stilbene, styrene, and isoquinoline scaffolds), they identified potent molecules (Figure 4B) that inhibited the formation of angiogenesis at a 100 µM concentration.

They also reported the development of a small molecule GAG mimetic selectively targeting colon CSCs [105]. They developed a tandem, dual-screen strategy to screen selectively for molecules targeting CSCs over the population of bulk cancer cells. Using this screening protocol and molecular dynamics-based algorithm, they showed that G2.2 (Figure 4C) is a structural and functional mimetic of HS06. G2.2 inhibited the growth of CSCs from multiple cell lines by induction of apoptosis and inhibition of self-renewal factors. G2.2 also inhibited the growth of tumor in CSC induced xenografts in vivo [106].

Another recent study highlighted the use of polyproline-based GAG mimetics (PGMs) that recapitulate key structural features of GAGs, including periodicity, the length of repeating units, turnability, and helicity, as shown through molecular dynamics simulations [107]. One of the synthesized PGMs, {Z}_12_ (Figure 4D), inhibited the interaction of CS-E with P-selectin, which is implicated in metastasis and inflammation. It also reduced metastasis in vivo as effectively as heparin and tinzaparin. Additionally, {Z}_12_ did not have inhibitory effects on the coagulation cascade enzymes, resulting in no/minimal bleeding side-effects. In animal models, it did not induce weight loss, elevate liver damage markers, or cause histopathological abnormalities, suggesting its safe use in vivo.

### 4.2. GAG Mimetics as Anti-Inflammatory Agents

#### 4.2.1. Saccharide-Based GAG Mimetics

**Figure 5:**
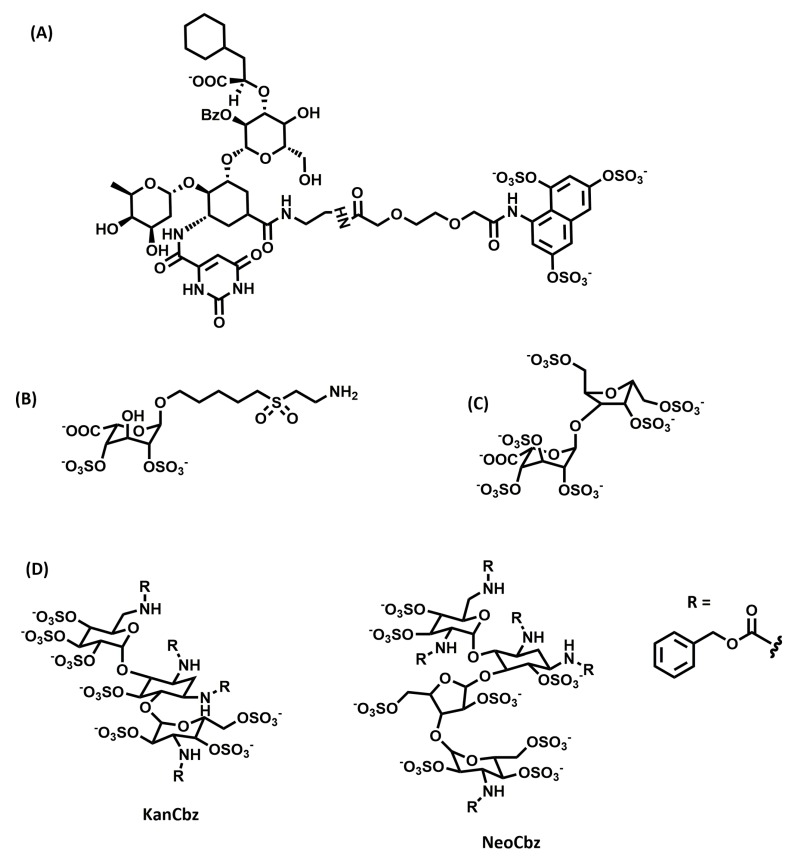
Chemical structures of saccharide glycosaminoglycan mimetics developed as anti-inflammatory agents: (**A**) GMI-1070, (**B**) 2,4-*O*-di-sulfated iduronic acid (Di-S-IdoA), (**C**) Hep- super-sulfated disaccharide (SSD), and (**D**) KanCbz and NeoCbz.

GlycoMimetics Inc. has developed a pan-selectin antagonist, GMI-1070 (Figure 5A), to treat vascular occlusions in people with sickle cell disease [108]. Recurrent vascular occlusions, as seen in sickle cell disease, lead to chronic inflammation and, eventually, irreversible organ damage. GMI-1070, when administered in mice, resulted in an increase in leukocyte rolling, which is indicative of selectin inhibition. It reduced leukocyte adherence to endothelium, inhibited red blood cell and leukocyte interactions, and also inhibited vascular occlusion. It was demonstrated to be safe in Phase I clinical trials [109], reduced the time to resolve vascular occlusive events in Phase II [110], and is currently in Phase III clinical trials.

Fukada and co-workers identified a synthetic, non-natural monosaccharide, 2,4-*O*-di-sulfated iduronic acid (Di-S-IdoA; Figure 5B), as an inhibitor of CCL20-HS interaction by screening a glycan array [111]. It also inhibited the binding of CXCL8 and L-selectin. The in vivo effects of the compound in mice models of allergy were tested via tail vein injection or nasal inhalation. Di-S-IdoA showed a reduction in leukocyte recruitment into the lungs, indicating a potential use of the compound to reduce the inflammatory response.

Another study showed the development of a super-sulfated disaccharide sequence of heparin (Hep-SSD; Figure 5C) from a non-anticoagulant inactive disaccharide [112]. Hep-SSD inhibited allergic airway responses in a sheep model of allergic asthma when given by both aerosol and oral administration routes in a dose-dependent manner.

Kerns and co-workers screened a panel of *N*-arylacyl-*O*-sulfated aminoglucosides against neutrophil serine proteases, including human neutrophil elastase, proteinase 3, and cathepsin G, which are upregulated in inflammatory lung conditions such as chronic obstructive pulmonary disease, cystic fibrosis, and acute lung injury [113]. They identified two lead compounds (Figure 5D), a kanamycin-based compound (KanCbz) and a neomycin-based compound (NeoCbz), as potent inhibitors of one or more of the proteases. The results demonstrated that these lead compounds can be further exploited for multi-target inhibitor drugs for attenuating inflammation mediated by proteases.

**Figure 6:**
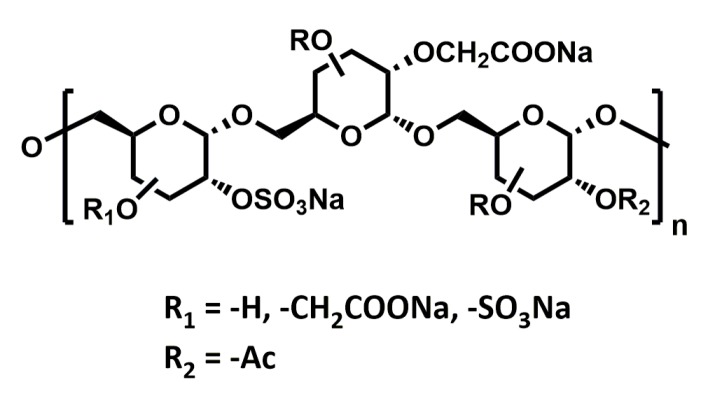
Chemical structure of RGTA^®^.

RGTA^®^s (ReGeneraTing Agents; Figure 6) are another class of HS mimetics that are designed to replace degraded HS in the ECM of injured tissue [114]. Chemically, they are polymers of dextran with ~250 glycan residues substituted with sulfates and carboxyl groups to mimic HS. In contrast to HS, which is made of β1-4 glycosidic bonds, RGTA^®^s are linked by α1-6 carbon-carbon bonds. This makes their structure resistant to enzymatic degradation (chondroitinase ABC, dextranase, hyaluronidase, and heparinases I, II, and III), helping them retain their structure and activity even in the presence of chronic wounds and inflammation. The ability of these compounds to improve wound healing has been intensively studied in numerous pathological conditions, and they are currently in the clinic for the treatment of chronic skin and corneal lesions.

#### 4.2.2. Non-Saccharide GAG Mimetics

**Figure 7:**
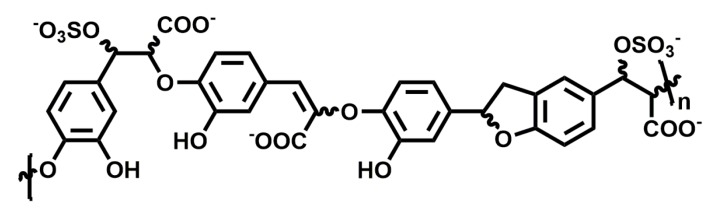
Chemical structure of CDSO_3_, *n* = 5–13.

The ability of sulfated low molecular weight lignins to inhibit inflammation caused by human neutrophil elastase, for the treatment of emphysema, was studied by Desai and co-workers. They found that sulfated caffeic acid (CDSO_3_; Figure 7) inhibited human neutrophil elastase in a concentration-dependent manner with an IC_50_ of 0.43 ± 0.04 µM and showed anti-oxidative and anti-inflammatory properties in vitro [115]. Furthermore, it attenuated the development of emphysema in rat models where emphysema was induced with human neutrophil elastase and cigarette smoke extract [116]. It was able to reduce inflammation in both a preventive and interventional manner when administered locally to the lung with doses as low as 30–100 µg/kg.

When endothelial lung cell death was induced in vitro using inhibitors for histone deacetylase and VEGF receptor, or through cigarette smoke, CDSO_3_ treatment significantly inhibited cell death when compared to control [117]. In a rat model with induced apoptotic emphysema, 60 µg/kg of CDSO_3_ produced significant blockade of lung damage and resulted in increased exercise endurance. Overall, CDSO_3_ appears to be promising in developing a novel treatment for emphysema.

## 5. Conclusions

In the past, the biological information of an organism was considered to be stored in the DNA, RNA, and proteins, and sugars were considered as a mere source of energy, lacking any other biological activities. Hence, the majority of research was focused on understanding the roles DNA, RNA, and proteins play in different physiological and pathological conditions. Furthermore, studies on glycans lagged far behind other macromolecules because of limited technology available to study their structural diversity and complexity. With advancements in mass spectrometry, lectin and antibody arrays, imaging technologies, glycan microarrays, and bioinformatic tools, it has now become possible to study the glycome, expanding the field of glycobiology.

Today, glycans are known to play diverse roles in an organism. GAGs, in particular, regulate a multitude of functions by interacting with proteins, cells, and tissues in the human body. They play a major role in almost every physiological activity in the body and are essential in maintaining homeostasis. Moreover, changes in their expression and/or the structure of GAGs are observed in multiple pathological conditions and are being employed as biomarkers for disease progression.

Since the discovery of heparin, more than 100 years ago, GAGs have been widely studied as therapeutics for cancer, inflammation, infections, lung diseases, etc. However, their success is being halted because of their inherent heterogeneity and affinity towards multiple targets, leading to side-effects. As a result of dealing with the limitations of GAGs, there seems to be a drift from heterogeneous GAGs derived from animal sources to homogenous, synthetic, or semi-synthetic GAG mimetics. These GAG mimetics not only offer advantages with homogeneity and synthetic feasibility, but also (usually) possess increased potency, selectivity, and better pharmacokinetic properties with lesser adverse effects. In conclusion, the field of glycosaminoglycan drug discovery is rapidly progressing with multiple GAGs/GAG mimetics already in the clinic. The next decade is going to see major changes in both the structural studies of GAGs and their corresponding role in therapy with more GAG-based drugs making their way to the clinic and rewriting the historical notion of GAGs in drug discovery.

## Figures and Tables

**Table 1:** Different classes of GAG-binding proteins and their biological activities.

Class of Binding Proteins	Physiological Function	Example
Enzymes	Coagulation	Factor Xa [4]
Enzyme inhibitors	Coagulation, inflammation	Antithrombin III [4]
Cell adhesion proteins	Cell adhesion, inflammation, metastasis	Platelet/endothelial cell adhesion molecule-1 [5]
Extracellular matrix (ECM) proteins	Cell adhesion, matrix organization	Annexin V [6]
Growth factors	Mitogenesis, cell migration	Fibroblast growth factor [7,8]
Chemokines	Chemotaxis, signaling inflammation	IL-8 [9]
Morphogens	Cell specification, tissue differentiation, development	BMP-2 [10]
Lipid-binding proteins	Lipid metabolism, cell membrane functions	Lipoprotein lipase [11]
Pathogen surface proteins	Pathogen infections	Circumsporozoite [12]
Viral proteins	Viral infections	Glycoprotein D [13]

## Abbreviations

GAG	Glycosaminoglycan
HA	Hyaluronan
CS	Chondroitin sulfate
DS	Dermatan sulfate
HS	Heparan sulfate
KS	Keratan sulfate
HSPG	Heparan sulfate proteoglycan
FGF2	Fibroblast growth factor 2
EMT	Epithelial to mesenchymal transition
FGFR-1	Fibroblast growth factor receptor-1
EGFR	Epidermal growth factor receptor
CSPG	Chondroitin sulfate proteoglycan
VEGF	Vascular endothelial growth factor
PDGF	Platelet-derived growth factor
MAPK	Mitogen-activated protein kinase
CSC	Cancer-stem cell
ODSH	2-*O*, 3-*O*-desulfated heparin

## References

[1] Lindahl U., Couchman J., Kimata K., Esko J.D., Varki A., Cummings R.D., Esko J.D., Freeze H.H., Stanley P., Bertozzi C.R., Hart G.W., Etzler M.E. (2009). Proteoglycans and sulfated glycosaminoglycans. Essentials of Glycobiology.

[2] Zhang L. (2010). Glycosaminoglycan (GAG) biosynthesis and GAG-binding proteins. Prog. Mol. Biol. Transl. Sci..

[3] Xu D., Esko J.D. (2014). Demystifying heparan sulfate-protein interactions. Annu. Rev. Biochem..

[4] Barbucci R., Magnani A., Lamponi S., Albanese A. (1996). Chemistry and biology of glycosaminoglycans in blood coagulation. Polym. Adv. Technol..

[5] DeLisser H.M., Yan H.C., Newman P.J., Muller W.A., Buck C.A., Albelda S.M. (1993). Platelet/endothelial cell adhesion molecule-1 (CD31)-mediated cellular aggregation involves cell surface glycosaminoglycans. J. Biol. Chem..

[6] Capila I., VanderNoot V.A., Mealy T.A., Seaton B.A., Linhardt R.J. (1999). Interaction of heparin with annexin V. FEBS Lett..

[7] Sanderson R.D. (2001). Heparan sulfate proteoglycans in invasion and metastasis. Semin. Cell Dev. Biol..

[8] Kure S., Yoshie O., Aso H. (1987). Metastatic potential of murine B16 melanoma correlates with reduced surface heparan sulfate glycosaminoglycan. Jpn. J. Cancer Res..

[9] Schlorke D., Thomas L., Samsonov S.A., Huster D., Arnhold J., Pichert A. (2012). The influence of glycosaminoglycans on IL-8-mediated functions of neutrophils. Carbohydr. Res..

[10] Miguez P.A., Terajima M., Nagaoka H., Mochida Y., Yamauchi M. (2011). Role of glycosaminoglycans of biglycan in BMP-2 signaling. Biochem. Biophys. Res. Commun..

[11] Olsson U., Ostergren-Lundén G., Moses J. (2001). Glycosaminoglycan-lipoprotein interaction. Glycoconj. J..

[12] Ying P., Shakibaei M., Patankar M.S., Clavijo P., Beavis R.C., Clark G.F., Frevert U. (1997). The malaria circumsporozoite protein: Interaction of the conserved regions I and II-plus with heparin-like oligosaccharides in heparan sulfate. Exp. Parasitol..

[13] Gangji R.N., Sankaranarayanan N.V., Elste J., Al-Horani R.A., Afosah D.K., Joshi R., Tiwari V., Desai U.R. (2018). Inhibition of herpes simplex virus-1 entry into human cells by nonsaccharide glycosaminoglycan mimetics. ACS Med. Chem. Lett..

[14] Rabenstein D.L. (2002). Heparin and heparan sulfate: Structure and function. Nat. Prod. Rep..

[15] Varki A., Cummings R.D., Esko J.D., Freeze H.H., Stanley P., Bertozzi C.R., Hart G.W., Etzler M.E. (2009). Essentials of Glycobiology.

[16] Afratis N., Gialeli C., Nikitovic D., Tsegenidis T., Karousou E., Theocharis A.D., Pavão M.S., Tzanakakis G.N., Karamanos N.K. (2012). Glycosaminoglycans: Key players in cancer cell biology and treatment. FEBS J..

[17] Volpi N. (2006). Therapeutic applications of glycosaminoglycans. Curr. Med. Chem..

[18] Theocharis A.D., Skandalis S.S., Tzanakakis G.N., Karamanos N.K. (2010). Proteoglycans in health and disease: Novel roles for proteoglycans in malignancy and their pharmacological targeting. FEBS J..

[19] Sasisekharan R., Shriver Z., Venkataraman G., Narayanasami U. (2002). Roles of heparan-sulphate glycosaminoglycans in cancer. Nat. Rev. Cancer.

[20] Nikitovic D., Assouti M., Sifaki M., Katonis P., Krasagakis K., Karamanos N.K., Tzanakakis G.N. (2008). Chondroitin sulfate and heparan sulfate-containing proteoglycans are both partners and targets of basic fibroblast growth factor-mediated proliferation in human metastatic melanoma cell lines. Int. J. Biochem. Cell Biol..

[21] Crespo A., García-Suárez O., Fernández-Vega I., Solis-Hernandez M.P., García B., Castañón S., Quirós L.M. (2018). Heparan sulfate proteoglycans undergo differential expression alterations in left sided colorectal cancer, depending on their metastatic character. BMC Cancer.

[22] Fernández-Vega I., García-Suárez O., García B., Crespo A., Astudillo A., Quirós L.M. (2015). Heparan sulfate proteoglycans undergo differential expression alterations in right sided colorectal cancer, depending on their metastatic character. BMC Cancer.

[23] Strutz F., Zeisberg M., Ziyadeh F.N., Yang C.Q., Kalluri R., Müller G.A., Neilson E.G., Renziehausen A., Sisic Z. (2002). Role of basic fibroblast growth factor-2 in epithelial-mesenchymal transformation. Kidney Int..

[24] Mundhenke C., Meyer K., Drew S., Friedl A. (2002). Heparan sulfate proteoglycans as regulators of fibroblast growth factor-2 receptor binding in breast carcinomas. Am. J. Pathol..

[25] Chalkiadaki G., Nikitovic D., Berdiaki A., Sifaki M., Krasagakis K., Katonis P., Karamanos N.K., Tzanakakis G.N. (2009). Fibroblast growth factor-2 modulates melanoma adhesion and migration through a syndecan-4-dependent mechanism. Int. J. Biochem. Cell Biol..

[26] Zittermann S.I., Capurro M.I., Shi W., Filmus J. (2010). Soluble glypican 3 inhibits the growth of hepatocellular carcinoma in vitro and in vivo. Int. J. Cancer.

[27] Sistla J.C., Morla S., Alabbas A.H.B., Kalathur R.C., Sharon C., Patel B.B., Desai U.R. (2019). Polymeric fluorescent heparin as one-step FRET substrate of human heparanase. Carbohydr. Polym..

[28] Sanderson R.D., Elkin M., Rapraeger A.C., Ilan N., Vlodavsky I. (2017). Heparanase regulation of cancer, autophagy and inflammation: New mechanisms and targets for therapy. FEBS J..

[29] Arvatz G., Weissmann M., Ilan N., Vlodavsky I. (2016). Heparanase and cancer progression: New directions, new promises. Hum. Vaccines Immunother..

[30] Cohen-Kaplan V., Jrbashyan J., Yanir Y., Naroditsky I., Ben-Izhak O., Ilan N., Doweck I., Vlodavsky I. (2012). Heparanase induces signal transducer and activator of transcription (STAT) protein phosphorylation: Preclinical and clinical significance in head and neck cancer. J. Biol. Chem..

[31] Ramani V.C., Yang Y., Ren Y., Nan L., Sanderson R.D. (2011). Heparanase plays a dual role in driving hepatocyte growth factor (HGF) signaling by enhancing HGF expression and activity. J. Biol. Chem..

[32] Vlodavsky I., Gross-Cohen M., Weissmann M., Ilan N., Sanderson R.D. (2018). Opposing functions of heparanase-1 and heparanase-2 in cancer progression. Trends Biochem. Sci..

[33] Khurana A., Liu P., Mellone P., Lorenzon L., Vincenzi B., Datta K., Yang B., Linhardt R.J., Lingle W., Chien J. (2011). HSulf-1 modulates FGF2- and hypoxia-mediated migration and invasion of breast cancer cells. Cancer Res..

[34] Peterson S.M., Iskenderian A., Cook L., Romashko A., Tobin K., Jones M., Norton A., Gómez-Yafal A., Heartlein M.W., Concino M.F. (2010). Human Sulfatase 2 inhibits in vivo tumor growth of MDA-MB-231 human breast cancer xenografts. BMC Cancer.

[35] Lemjabbar-Alaoui H., Van Zante A., Singer M.S., Xue Q., Wang Y.Q., Tsay D., He B., Jablons D.M., Rosen S.D. (2010). Sulf-2, a heparan sulfate endosulfatase, promotes human lung carcinogenesis. Oncogene.

[36] Mikami T., Kitagawa H. (2013). Biosynthesis and function of chondroitin sulfate. Biochim. Biophys. Acta.

[37] Ricciardelli C., Brooks J.H., Suwiwat S., Sakko A.J., Mayne K., Raymond W.A., Seshadri R., LeBaron R.G., Horsfall D.J. (2002). Regulation of stromal versican expression by breast cancer cells and importance to relapse-free survival in patients with node-negative primary breast cancer. Clin. Cancer Res..

[38] Ricciardelli C., Mayne K., Sykes P.J., Raymond W.A., McCaul K., Marshall V.R., Horsfall D.J. (1998). Elevated levels of versican but not decorin predict disease progression in early-stage prostate cancer. Clin. Cancer Res..

[39] Skandalis S.S., Kletsas D., Kyriakopoulou D., Stavropoulos M., Theocharis D.A. (2006). The greatly increased amounts of accumulated versican and decorin with specific post-translational modifications may be closely associated with the malignant phenotype of pancreatic cancer. Biochim. Biophys. Acta.

[40] Yang J., Price M.A., Gui Y.L., Bar-Eli M., Salgia R., Jagedeeswaran R., Carlson J.H., Ferrone S., Turley E.A., McCarthy J.B. (2009). Melanoma proteoglycan modifies gene expression to stimulate tumor cell motility, growth, and epithelial-to-mesenchymal transition. Cancer Res..

[41] Theocharis A.D., Tsolakis I., Tzanakakis G.N., Karamanos N.K. (2006). Chondroitin Sulfate as a Key Molecule in the Development of Atherosclerosis and Cancer Progression. Adv. Pharmacol..

[42] Ten Dam G.B., Van De Westerlo E.M.A., Purushothaman A., Stan R.V., Bulten J., Sweep F.C.G.J., Massuger L.F., Sugahara K., Van Kuppevelt T.H. (2007). Antibody GD3G7 selected against embryonic glycosaminoglycans defines chondroitin sulfate-E domains highly up-regulated in ovarian cancer and involved in vascular endothelial growth factor binding. Am. J. Pathol..

[43] Li F., Ten Dam G.B., Murugan S., Yamada S., Hashiguchi T., Mizumoto S., Oguri K., Okayama M., Van Kuppevelt T.H., Sugahara K. (2008). Involvement of highly sulfated chondroitin sulfate in the metastasis of the Lewis lung carcinoma cells. J. Biol. Chem..

[44] Basappa, Murugan S., Sugahara K.N., Lee C.M., ten Dam G.B., Van Kuppevelt T.H., Miyasaka M., Yamada S., Sugahara K. (2009). Involvement of chondroitin sulfate E in the liver tumor focal formation of murine osteosarcoma cells. Glycobiology.

[45] Fthenou E., Zafiropoulos A., Tsatsakis A., Stathopoulos A., Karamanos N.K., Tzanakakis G.N. (2006). Chondroitin sulfate A chains enhance platelet derived growth factor-mediated signalling in fibrosarcoma cells. Int. J. Biochem. Cell Biol..

[46] Fthenou E., Zong F., Zafiropoulos A., Dobra K., Hjerpe A., Tzanakakis G.N. (2009). Chondroitin sulfate A regulates fibrosarcoma cell adhesion motility and migration through JNK and tyrosine kinase signaling pathways. In Vivo.

[47] Auvinen P., Tammi R., Parkkinen J., Tammi M., Ågren U., Johansson R., Hirvikoski P., Eskelinen M., Kosma V.M. (2000). Hyaluronan in peritumoral stroma and malignant cells associates with breast cancer spreading and predicts survival. Am. J. Pathol..

[48] Pirinen R., Tammi R., Tammi M., Hirvikoski P., Parkkinen J.J., Johansson R., Böhm J., Hollmén S., Kosma V.M. (2001). Prognostic value of hyaluronan expression in non-small-cell lung cancer: Increased stromal expression indicates unfavorable outcome in patients with adenocarcinoma. Int. J. Cancer.

[49] Anttila M.A., Tammi R.H., Tammi M.I., Syrjänen K.J., Saarikoski S.V., Kosma V.M. (2000). High levels of stromal hyaluronan predict poor disease outcome in epithelial ovarian cancer. Cancer Res..

[50] Tammi R.H., Passi A.G., Rilla K., Karousou E., Vigetti D., Makkonen K., Tammi M.I. (2011). Transcriptional and post-translational regulation of hyaluronan synthesis. FEBS J..

[51] Kosunen A., Ropponen K., Kellokoski J., Pukkila M., Virtaniemi J., Valtonen H., Kumpulainen E., Johansson R., Tammi R., Tammi M. (2004). Reduced expression of hyaluronan is a strong indicator of poor survival in oral squamous cell carcinoma. Oral Oncol..

[52] Karjalainen J.M., Tammi R.H., Tammi M.I., Eskelinen M.J., Ågren U.M., Parkkinen J.J., Alhava E.M., Kosma V.M. (2000). Reduced level of CD44 and hyaluronan associated with unfavorable prognosis in clinical stage I cutaneous melanoma. Am. J. Pathol..

[53] Toole B.P. (2004). Hyaluronan: From extracellular glue to pericellular cue. Nat. Rev. Cancer.

[54] Bourguignon L.Y.W., Singleton P.A., Zhu H., Diedrich F. (2003). Hyaluronan-mediated CD44 interaction with RhoGEF and Rho kinase promotes Grb2-associated binder-1 phosphorylation and phosphatidylinositol 3-kinase signaling leading to cytokine (macrophage-colony stimulating factor) production and breast tumor progressio. J. Biol. Chem..

[55] Ponta H., Sherman L., Herrlich P.A. (2003). CD44: From adhesion molecules to signalling regulators. Nat. Rev. Mol. Cell. Biol..

[56] Okamoto I., Tsuiki H., Kenyon L.C., Godwin A.K., Emlet D.R., Holgado-Madruga M., Lanham I.S., Joynes C.J., Vo K.T., Guha A. (2002). Proteolytic cleavage of the CD44 adhesion molecule in multiple human tumors. Am. J. Pathol..

[57] Sugahara K.N., Murai T., Nishinakamura H., Kawashima H., Saya H., Miyasaka M. (2003). Hyaluronan oligosaccharides induce CD44 cleavage and promote cell migration in CD44-expressing tumor cells. J. Biol. Chem..

[58] Borsig L. (2010). Heparin as an inhibitor of cancer progression. Prog. Mol. Biol. Transl. Sci..

[59] Bochenek J., Püsküllüoglu M., Krzemieniecki K. (2013). The antineoplastic effect of low-molecular-weight heparins-A literature review. Contemp. Oncol..

[60] Zhou H., Roy S., Cochran E., Zouaoui R., Chu C.L., Duffner J., Zhao G., Smith S., Galcheva-Gargova Z., Karlgren J. (2011). M402, a novel Heparan sulfate mimetic, targets multiple pathways implicated in tumor progression and metastasis. PLoS ONE.

[61] Ritchie J.P., Ramani V.C., Ren Y., Naggi A., Torri G., Casu B., Penco S., Pisano C., Carminati P., Tortoreto M. (2011). SST0001, a chemically modified heparin, inhibits myeloma growth and angiogenesis via disruption of the heparanase/syndecan-1 axis. Clin. Cancer Res..

[62] Norrby K., Nordenhem A. (2010). Dalteparin, a low-molecular-weight heparin, promotes angiogenesis mediated by heparin-binding VEGF-A in vivo. APMIS.

[63] Gomes A.M., Kozlowski E.O., Borsig L., Teixeira F.C.O.B., Vlodavsky I., Pavão M.S.G. (2015). Antitumor properties of a new non-anticoagulant heparin analog from the mollusk Nodipecten nodosus: Effect on P-selectin, heparanase, metastasis and cellular recruitment. Glycobiology.

[64] Long A., Chu C.L., Galcheva-Gargova Z., Holte K., Duffner J., Schultes B.C. (2017). Role of M402, a novel heparan sulfate mimetic, in pancreatic cancer cell invasion and metastasis: Inhibition of the Sonic Hedgehog pathway and heparanase activity. J. Clin. Oncol..

[65] Patel N.J., Sharon C., Baranwal S., Boothello R.S., Desai U.R., Patel B.B., Patel N.J., Sharon C., Baranwal S., Boothello R.S. (2016). Heparan sulfate hexasaccharide selectively inhibits cancer stem cells self-renewal by activating p38 MAP kinase. Oncotarget.

[66] Liu D., Shriver Z., Venkataraman G., Shabrawi Y.E., Sasisekharan R. (2002). Tumor cell surface heparan sulfate as cryptic promoters or inhibitors of tumor growth and metastasis. Proc. Natl. Acad. Sci. USA.

[67] Borsig L., Wang L., Cavalcante M.C.M., Cardilo-Reis L., Ferreira P.L., Mourão P.A.S., Esko J.D., Pavão M.S.G. (2007). Selectin blocking activity of a fucosylated chondroitin sulfate glycosaminoglycan from sea cucumber: Effect on tumor metastasis and neutrophil recruitment. J. Biol. Chem..

[68] Liu Y., Yang H., Otaka K., Takatsuki H., Sakanishi A. (2005). Effects of vascular endothelial growth factor (VEGF) and chondroitin sulfate a on human monocytic THP-1 cell migration. Colloids Surf. B Biointerfaces.

[69] Pumphrey C.Y., Theus A.M., Li S., Parrish R.S., Sanderson R.D. (2002). Neoglycans, carbodiimide-modified glycosaminoglycans: A new class of anticancer agents that inhibit cancer cell proliferation and induce apoptosis. Cancer Res..

[70] Moore K.L., Patel K.D., Bruehl R.E., Fugang L., Johnson D.A., Lichenstein H.S., Cummings R.D., Bainton D.F., McEver R.P. (1995). P-selectin glycoprotein ligand-1 mediates rolling of human neutrophils on P-selectin. J. Cell Biol..

[71] Parish C.R. (2006). The role of heparan sulphate in inflammation. Nat. Rev. Immunol..

[72] Webb L.M., Ehrengruber M.U., Clark-Lewis I., Baggiolini M., Rot A. (1993). Binding to heparan sulfate or heparin enhances neutrophil responses to interleukin 8. Proc. Natl. Acad. Sci. USA.

[73] Gotte M. (2003). Syndecans in inflammation. FASEB J..

[74] Theoharides T.C., Alysandratos K.-D., Angelidou A., Delivanis D.-A., Sismanopoulos N., Zhang B., Asadi S., Vasiadi M., Weng Z., Miniati A. (2012). Mast cells and inflammation. Biochim. Biophys. Acta.

[75] Oschatz C., Maas C., Lecher B., Jansen T., Björkqvist J., Tradler T., Sedlmeier R., Burfeind P., Cichon S., Hammerschmidt S. (2011). Mast Cells Increase Vascular Permeability by Heparin-Initiated Bradykinin Formation In Vivo. Immunity.

[76] Tyrrell D.J., Horne A.P., Holme K.R., Preuss J.M.H., Page C.P. (1999). Heparin in Inflammation: Potential Therapeutic Applications beyond Anticoagulation. Adv. Pharmacol..

[77] Ahmed T., Garrigo J., Danta I. (1993). Preventing bronchoconstriction in exercise-induced asthma with inhaled heparin. N. Engl. J. Med..

[78] Zezos P., Papaioannou G., Nikolaidis N., Patsiaoura K., Papageorgiou A., Vassiliadis T., Giouleme O., Evgenidis N. (2006). Low-molecular-weight heparin (enoxaparin) as adjuvant therapy in the treatment of active ulcerative colitis: A randomized, controlled, comparative study. Aliment. Pharmacol. Ther..

[79] Saliba M.J. (2001). Heparin in the treatment of burns: A review. Burns.

[80] Mousavi S., Moradi M., Khorshidahmad T., Motamedi M. (2015). Anti-inflammatory effects of heparin and its derivatives: A systematic review. Adv. Pharmacol. Sci..

[81] Griffin K.L., Fischer B.M., Kummarapurugu A.B., Zheng S., Kennedy T.P., Rao N.V., Foster W.M., Voynow J.A. (2014). 2-*O*, 3-*O*-desulfated heparin inhibits neutrophil elastase-induced HMGB-1 secretion and airway inflammation. Am. J. Respir. Cell Mol. Biol..

[82] Brito A.S., Arimatéia D.S., Souza L.R., Lima M.A., Santos V.O., Medeiros V.P., Ferreira P.A., Silva R.A., Ferreira C.V., Justo G.Z. (2008). Anti-inflammatory properties of a heparin-like glycosaminoglycan with reduced anti-coagulant activity isolated from a marine shrimp. Bioorganic Med. Chem..

[83] Kozlowski E.O., Pavao M.S.G., Borsig L. (2011). Ascidian dermatan sulfates attenuate metastasis, inflammation and thrombosis by inhibition of P-selectin. J. Thromb. Haemost..

[84] Cañas N., Gorina R., Planas A.M., Vergés J., Montell E., García A.G., López M.G. (2010). Chondroitin sulfate inhibits lipopolysaccharide-induced inflammation in rat astrocytes by preventing nuclear factor kappa B activation. Neuroscience.

[85] Caterson B., Melrose J. (2018). Keratan sulfate, a complex glycosaminoglycan with unique functional capability. Glycobiology.

[86] Hayashi M., Kadomatsu K., Ishiguro N. (2010). Keratan sulfate suppresses cartilage damage and ameliorates inflammation in an experimental mice arthritis model. Biochem. Biophys. Res. Commun..

[87] Winsz-Szczotka K., Komosińska-Vassev K., Kuźnik-Trocha K., Siwiec A., Zegleń B., Olczyk K. (2015). Circulating keratan sulfate as a marker of metabolic changes of cartilage proteoglycan in juvenile idiopathic arthritis; Influence of growth factors as well as proteolytic and prooxidative agents on aggrecan alterations. Clin. Chem. Lab. Med..

[88] Carlson E.C., Lin M., Liu C.Y., Kao W.W., Perez V.L., Pearlman E. (2007). Keratocan and lumican regulate neutrophil infiltration and corneal clarity in lipopolysaccharide-induced keratitis by direct interaction with CXCL1. J. Biol. Chem..

[89] Carlson E.C., Sun Y., Auletta J., Kao W.W.-Y., Liu C.-Y., Perez V.L., Pearlman E. (2010). Regulation of corneal inflammation by neutrophil-dependent cleavage of keratan sulfate proteoglycans as a model for breakdown of the chemokine gradient. J. Leukoc. Biol..

[90] Shirato K., Gao C., Ota F., Angata T., Shogomori H., Ohtsubo K., Yoshida K., Lepenies B., Taniguchi N. (2013). Flagellin/Toll-like receptor 5 response was specifically attenuated by keratan sulfate disaccharide via decreased EGFR phosphorylation in normal human bronchial epithelial cells. Biochem. Biophys. Res. Commun..

[91] Gao C., Fujinawa R., Yoshida T., Ueno M., Ota F., Kizuka Y., Hirayama T., Korekane H., Kitazume S., Maeno T. (2017). A keratan sulfate disaccharide prevents inflammation and the progression of emphysema in murine models. Am. J. Physiol. Lung Cell Mol. Physiol..

[92] Mohamed S., Coombe D.R. (2017). Heparin mimetics: Their therapeutic potential. Pharmaceuticals.

[93] Shojania A.M., Tetreault J., Turnbull G. (1988). The variations between heparin sensitivity of different lots of activated partial thromboplastin time reagent produced by the same manufacturer. Am. J. Clin. Pathol..

[94] Jones C.J., Beni S., Limtiaco J.F., Langeslay D.J., Larive C.K. (2011). Heparin characterization: Challenges and solutions. Annu. Rev. Anal. Chem..

[95] Hedlund K.D., Coyne D.P., Sanford D.M., Huddelson J. (2013). The heparin recall of 2008. Perfusion.

[96] Deasi U.R. (2013). The promise of sulfated synthetic small molecules as modulators of glycosaminoglycanfunction. Future Med. Chem..

[97] Khachigian Levon M., Parish Christopher R. (2004). Phosphomannopentaose sulfate (PI-88): Heparan sulfate mimetic with clinical potential in multiple vascular pathologies. Cardiovasc. Drug Rev..

[98] Lanzi C., Cassinelli G. (2018). Heparan sulfate mimetics in cancer therapy: The challenge to define structural determinants and the relevance of targets for optimal activity. Molecules.

[99] Dredge K., Hammond E., Davis K., Li C.P., Liu L., Johnstone K., Handley P., Wimmer N., Gonda T.J., Gautam A. (2010). The PG500 series: Novel heparan sulfate mimetics as potent angiogenesis and heparanase inhibitors for cancer therapy. Investig. New Drugs.

[100] Dredge K., Hammond E., Handley P., Gonda T.J., Smith M.T., Vincent C., Brandt R., Ferro V., Bytheway I. (2011). PG545, a dual heparanase and angiogenesis inhibitor, induces potent anti-tumour and anti-metastatic efficacy in preclinical models. Br. J. Cancer.

[101] Hammond E., Handley P., Dredge K., Bytheway I. (2013). Mechanisms of heparanase inhibition by the heparan sulfate mimetic PG545 and three structural analogues. FEBS Open Biol..

[102] Kuhnast B., El Hadri A., Boisgard R., Hinnen F., Richard S., Caravano A., Nancy-Portebois V., Petitou M., Tavitian B., Dollé F. (2016). Synthesis, radiolabeling with fluorine-18 and preliminary in vivo evaluation of a heparan sulphate mimetic as potent angiogenesis and heparanase inhibitor for cancer applications. Org. Biomol. Chem..

[103] Freeman C., Liu L., Banwell M.G., Brown K.J., Bezos A., Ferro V., Parish C.R. (2005). Use of sulfated linked cyclitols as heparan sulfate mimetics to probe the heparin/heparan sulfate binding specificity of proteins. J. Biol. Chem..

[104] Raman K., Karuturi R., Swarup V.P., Desai U.R., Kuberan B. (2012). Discovery of novel sulfonated small molecules that inhibit vascular tube formation. Bioorg. Med. Chem. Lett..

[105] Patel N.J., Karuturi R., Al-Horani R.A., Baranwal S., Patel J., Desai U.R., Patel B.B. (2014). Synthetic, non-saccharide, glycosaminoglycan mimetics selectively target colon cancer stem cells. ACS Chem. Biol..

[106] Boothello R.S., Patel N.J., Sharon C., Abdelfadiel E.I., Morla S., Brophy D.F., Lippman H.R., Desai U.R., Patel B.B. (2018). A Unique Nonsaccharide Mimetic of Heparin Hexasaccharide Inhibits Colon Cancer Stem Cells via p38 MAP Kinase Activation. Mol. Cancer Ther..

[107] Lim T.C., Cai S., Huber R.G., Bond P.J., Siew Chia P.X., Khou S.L., Gao S., Lee S.S., Lee S.G. (2018). Facile saccharide-free mimetics that recapitulate key features of glycosaminoglycan sulfation patterns. Chem. Sci..

[108] Chang J., Patton J.T., Sarkar A., Ernst B., Magnani J.L., Frenette P.S. (2010). GMI-1070, a novel pan-selectin antagonist, reverses acute vascular occlusions in sickle cell mice. Blood.

[109] Wun T., Styles L., DeCastro L., Telen M.J., Kuypers F., Cheung A., Kramer W., Flanner H., Rhee S., Magnani J.L. (2014). Phase 1 study of the E-selectin inhibitor GMI 1070 in patients with sickle cell anemia. PLoS ONE.

[110] Telen M.J., Wun T., McCavit T.L., De Castro L.M., Krishnamurti L., Lanzkron S., Hsu L.L., Smith W.R., Rhee S., Magnani J.L. (2015). Randomized phase 2 study of GMI-1070 in SCD: Reduction in time to resolution of vaso-occlusive events and decreased opioid use. Blood.

[111] Nonaka M., Gotze S., Seeberger P.H., Kononov A., Bao X., Fukuda M., Matsumura F., Broide D.H., Kandasamy J., Nakayama J. (2014). Synthetic di-sulfated iduronic acid attenuates asthmatic response by blocking T-cell recruitment to inflammatory sites. Proc. Natl. Acad. Sci. USA.

[112] Ahmed T., Smith G., Abraham W.M. (2014). Heparin-derived supersulfated disaccharide inhibits allergic airway responses in sheep. Pulm. Pharmacol. Ther..

[113] Craciun I., Fenner A.M., Kerns R.J. (2016). *N*-Arylacyl *O*-sulfonated aminoglycosides as novel inhibitors of human neutrophil elastase, cathepsin G and proteinase 3. Glycobiology.

[114] Barritault D., Gilbert-Sirieix M., Rice K.L., Siñeriz F., Papy-Garcia D., Baudouin C., Desgranges P., Zakine G., Saffar J.L., van Neck J. (2017). RGTA^®^ or ReGeneraTing Agents mimic heparan sulfate in regenerative medicine: From concept to curing patients. Glycoconj. J..

[115] Saluja B., Thakkar J.N., Li H., Desai U.R., Sakagami M. (2013). Novel low molecular weight lignins as potential anti-emphysema agents: In vitro triple inhibitory activity against elastase, oxidation and inflammation. Pulm. Pharmacol. Ther..

[116] Saluja B., Li H., Desai U.R., Voelkel N.F., Sakagami M. (2014). Sulfated caffeic acid dehydropolymer attenuates elastase and cigarette smoke extract-induced emphysema in rats: Sustained activity and a need of pulmonary delivery. Lung.

[117] Truong T.M., Li H., Dhapare S., Desai U.R., Voelkel N.F., Sakagami M. (2017). Sulfated dehydropolymer of caffeic acid: In vitro anti-lung cell death activity and in vivo intervention in emphysema induced by VEGF receptor blockade. Pulm. Pharmacol. Ther..

